# Application of Balloon AngioplaSty with the dIstal protection of Stent Retriever (BASIS) technique for acute intracranial artery atherosclerosis-related occlusion

**DOI:** 10.3389/fneur.2022.1049543

**Published:** 2022-11-17

**Authors:** Ting-yu Yi, Yan-min Wu, Ding-lai Lin, Zhi-nan Pan, Xiu-fen Zheng, Ji Gan, Mei-hua Wu, Xiao-hui Lin, Rong-cheng Chen, Li-san Zeng, Wen-huo Chen

**Affiliations:** Zhangzhou Affiliated Hospital of Fujian Medical University, Zhangzhou, China

**Keywords:** intracranial atherosclerosis, large vessel occlusion, BASIS, endovascular therapy, first pass effect

## Abstract

**Background:**

Endovascular therapy (EVT) is complex in the context of intracranial atherosclerosis (ICAS)-related large vessel occlusion (LVO) and the re-occlusion rates are high due to residual stenosis, the procedure time is long and the optimal EVT technique is unclear. The Balloon AngioplaSty with the dIstal protection of Stent Retriever (BASIS) technique is a novel thrombectomy technique that allows emergent balloon angioplasty to be performed *via* the wire of the retrieval stent. Our study presents our initial experience with the BASIS technique in ICAS-related LVO and assesses its feasibility.

**Method:**

In patients with ICAS-related LVO treated with BASIS, clinical and angiographic data were retrospectively analyzed. Angiographic data included first-pass reperfusion (PFR), the rate of residual stenosis, distal emboli, and re-occlusion post-procedure. The Extended Thrombolysis in Cerebral Infarction (eTICI) scale was used to assess reperfusion extent, and an eTICI score ≥2b was defined as successful perfusion. Clinical outcome was evaluated at 3 months (modified Rankin score [mRS]), and an mRS ≤ 2 was defined as a good clinical outcome.

**Results:**

A total of seven patients with ICAS-related LVO were included, and the median age of the patients was 76 years. All patients achieved eTICI 3 reperfusion and FPR. The residual stenosis rate ranged from 5 to 10%. None of the patients had re-occlusion post-procedure. The median puncture-to-reperfusion time was 51 min. None of the patients had a symptomatic cerebral hemorrhage, re-occlusion, distal embolism, and dissection. Good clinical outcomes were observed in four patients (4/7, 57.1%), and 1 patient (1/7, 14.3%) died.

**Conclusion:**

The BASIS technique is feasible and safe for treating acute ICAS-related LVO.

## Introduction

Endovascular thrombectomy (EVT) is the standard and effective treatment for ischemic stroke patients with large vessel occlusion (LVO) (1–8). The main strategy of EVT for LVO includes stentriever thrombectomy, aspiration thrombectomy, and emergent angioplasty. The optimal EVT therapy for LVO caused by intracranial atherosclerosis (ICAS)-related LVO is unclear. In contrast to embolic lesions, ICAS responds less to stentriever thrombectomy (9) and requires a more complex, technically demanding recanalization strategy and longer procedure time (10). The EVT strategy is complex in ICAS lesions due to the multiformity of plaque, angioarchitecture, and thrombus composition in such cases. The dilemma in treating ICAS-related LVO is listed as follows: (1) If stentriever thrombectomy is chosen as the first-line strategy, a thrombus located distal to the stenotic site is hard to retrieve by the retrieval stent for tight stenosis at the proximal segment, and artery dissection may also occur following repeated stentriever thrombectomy; (2) If emergent angioplasty is chosen as the first-line strategy, a thrombus located distal to the stenotic site will migrate distal to the stenotic site when the proximal stenotic site is opened, which will increase the difficulty and risk in treating it *via* EVT. On the other hand, the perforating artery may be occluded by thrombi caused by emergent angioplasty. Furthermore, the procedure-related complication rate might increase with additional endovascular operations (11, 12).

The dilemma may be resolved if angioplasty can be performed *via* balloon advancement through the wire of the retrieval stent. Therefore, we propose the novel EVT technique, which is called the BASIS technique (Balloon AngioplaSty with the dIstal protection of Stent Retriever), in treating ICAS-related LVO and assessed the feasibility of the BASIS technique in treating such types of lesions.

## Materials and methods

### Patients

Informed consent for treatment was obtained from all of the patients or their relatives. The study was approved by and conducted in accordance with the guidelines of our institutional review board (ID 2021 LWB227), and informed consent was not obtained for the study because of its retrospective nature. Patients with ICAS-related LVO who received the BASIS technique were included. Patients were selected based on the following criteria: (1) digital subtraction angiography (DSA) documentation of LVO; (2) ICAS was suspected by clinical presentation and the appearance of a tapered sign observed on CT angiography (CTA)/DSA (13) and the phenomenon of the “microcatheter first pass effect” (14) or culprit intracranial artery stenosis was diagnosed before stroke onset; and (3) the BASIS technique was adopted.

### Endovascular treatment (BASIS)

An endovascular procedure was performed under conscious sedation. An 8F sheath was retrogradely inserted into the femoral artery, and diagnostic angiography was performed to confirm LVO suspected to be caused by ICAS. The novel technique referred to as BASIS is first described in this study. In brief, after deployment of the guiding catheter into the ICA or vertebral artery (VA), the microcatheter and a microwire are navigated through the area of total occlusion to the distal patent artery, and the microcatheter is then retrieved on the proximal side of the thrombus. Angiography is performed with a guiding catheter to determine whether blood flows through the vessel at the site of occlusion. When such flow is observed, a microcatheter “first-pass effect” is verified, which indicates a high probability of ICAS-related LVO. If thrombi located distal to the stenotic site are observed during the microcatheter “first pass effect” test, the BASIS technique is strongly recommended.

**Figure 1:**
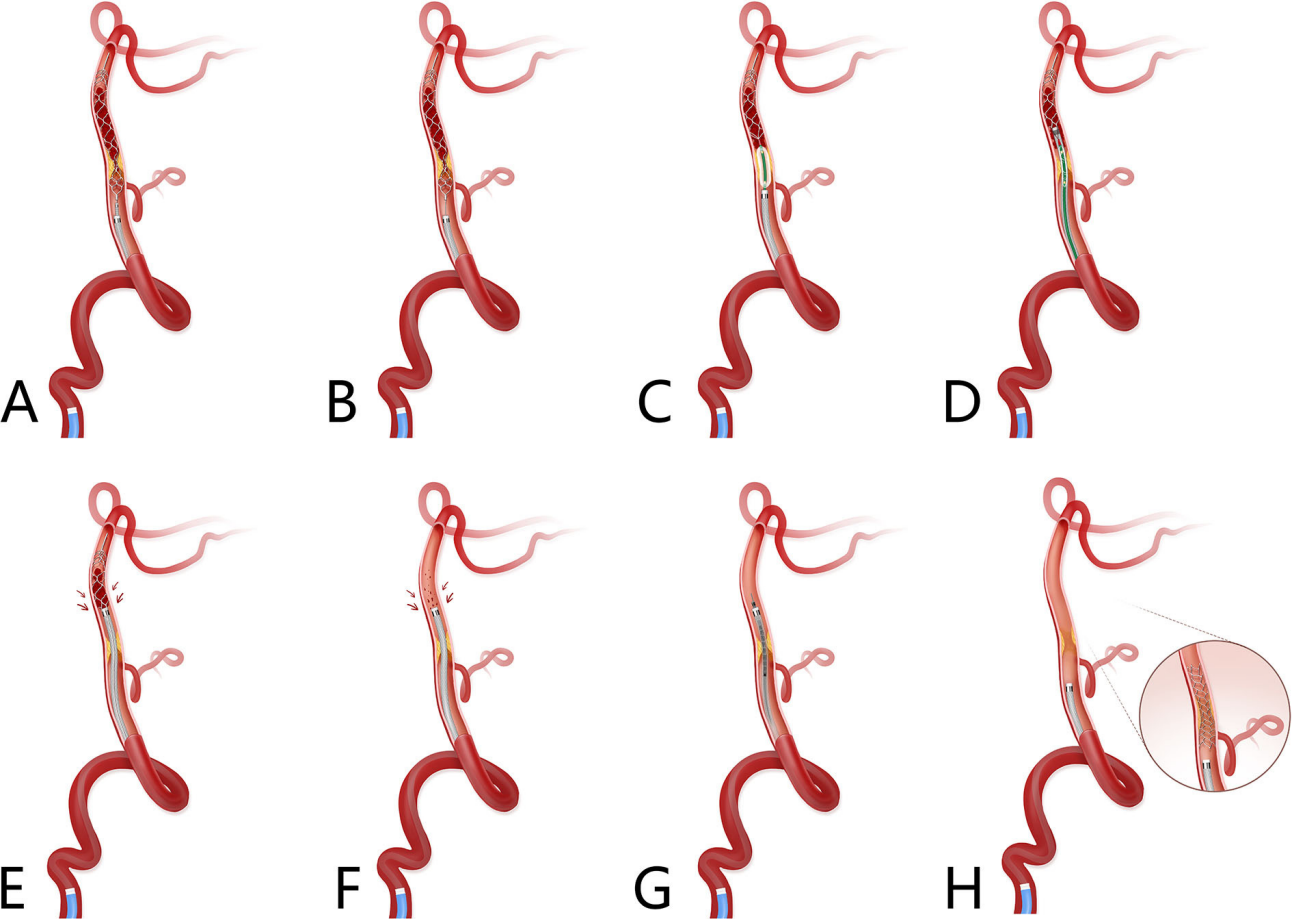
Schematic diagram of the Balloon AngioplaSty with the dIstal protection of Stent retriever (BASIS) technique. Basilar artery ICAS-related occlusion with thrombus formed distal to the stenotic site. **(A)** A microcatheter was navigated through the area of total occlusion to the distal patent artery, microcatheter first pass effect was performed to ascertain ICAS-related occlusion; microwire was retrieved while microcatheter stay at distal of occlusion site and retrieval stent, of which the diameter of wire was 0.014 inch, was deployed at the occlusion site, which fully covered the lesion and thrombus. **(B)** Microcatheter was withdrawn while stent was in place. **(C)** A suitable balloon was advanced into the stenotic site through wire of retrieval stent, and the stent was partially retrieved; then the balloon was inflated. **(D)** Aspiration catheter was inserted through the stenotic site when the balloon was deflated. **(E)** The stent retriever was withdrawn under continuous manual negative pressure exerted with a 50-ml locked syringe without changing the position of the aspiration catheter. **(F)** Continuous aspiration was performed while totally withdrawing the stent retriever and there was blood flow from the aspiration catheter. **(G)** Stent retriever was redeployed at the lesion site; then aspiration catheter was retrieved to the proximal site under continuous aspiration *via* 50-ml locked syringe. **(H)** Angiography was performed to determine the stenosis grade and reperfusion grade. If a successful reperfusion grade could be maintained, the stent was retrieved *via* a microcatheter and withdrawn, if successful reperfusion could not be achieved and/or residual stenosis was high, angioplasty *via* balloon could be reperformed, in some clinical scenarios of stent implantation. ICAS, indicates intracranial atherosclerosis.

A microcatheter was re-navigated through the area of total occlusion to the distal patent artery, microwire retrieved, and a retrieval stent of which the diameter of the wire was 0.014 inch, was deployed at the occlusion site, fully covering the lesion and thrombus (Figure 1A); the microcatheter was withdrawn while the stent was in place (Figure 1B); a suitable balloon was advanced into the stenotic site through the wire of the retrieval stent, the stent was partially retrieved, and the balloon inflated (Figure 1C); the aspiration catheter was inserted through the stenotic site while the balloon was deflated (Figure 1D); the stent retriever was withdrawn under continuous manual negative pressure formed with a 50-ml locked syringe without changing the position of the aspiration catheter (Figure 1E); continuous aspiration was performed *via* 50-ml locked syringe while totally withdrawing the stent retriever until there was blood flow from the aspiration catheter (Figure 1F); the retrieval stent was redeployed at the lesion site, then the aspiration catheter retrieved to the proximal site under continuous aspiration *via* 50-ml locked syringe (Figure 1G). Angiography was performed to determine the stenosis grade and reperfusion grade. If the successful reperfusion grade could be maintained, then the stent was retrieved *via* a microcatheter and withdrawn. If successful reperfusion could not be achieved and/or residual stenosis was high, angioplasty *via* balloon could be reperformed, in some clinical scenarios of stent implantation (Figure 1H).

This procedure was performed as per the following steps: (1) retrieval stent placement; (2) balloon angioplasty *via* wire of retrieval stent; (3) passage of aspiration catheter following after balloon deflated; (4) negative aspiration; (5) retrieval stent re-placement; (6) withdraw the retrieval stent; and (6) revascularization. The core novelty of this technique is summarized as Balloon AngioplaSty with the dIstal protection of a Stent Retriever; therefore, this technique, as adopted by us, is named the BASIS technique.

### Clinical and outcomes assessment and measures

The National Institutes of Health Stroke Scale (NIHSS) was used to assess patients' neurological function, and 90-day mRS was used to assess the clinical outcome. The primary clinical efficacy outcome was the rate of good prognosis at 90 days postprocedure, as defined by an mRS score of 0–2. Safety was evaluated by the incidence of symptomatic intracranial hemorrhage (sICH) and the occurrence of re-occlusion post-procedure, which was assessed by CTA performed 24–72 h post-procedure. High-resolution MR was performed in high-cooperation patients, sICH was defined according to the Heidelberg criteria (15).

### Angiographic and procedural outcomes

Brain tissue reperfusion was assessed radiologically immediately after the operation by the Extended Thrombolysis in Cerebral Infarction (eTICI) scale, with successful reperfusion defined as an eTICI score ≥2b (16). Achievement of complete reperfusion (eTICI 3) after a single operation of EVT was called first-pass reperfusion, which is the first-pass effect (FPE) (17). The primary angiographic outcomes were the rate of successful reperfusion, the FPE rate, and the residual stenosis rate. Secondary outcome measures included the incidence of distal emboli, arterial dissection, and procedure-related complications.

### Data availability

Access to patient records for data collection and analysis was approved by our local medical ethics committee, and informed consent was not obtained because of the retrospective nature of the study. We will share the identified data of participants in our study upon request.

### Statistical analysis

The data for categorical variables are described in absolute and relative frequencies. The data for continuous variables are given as the median and range or the mean and standard deviation. All statistical analyses were performed with IBM SPSS Statistics 22.0 (IBM, Inc., Armonk, NY).

## Results

**Figure 2:**
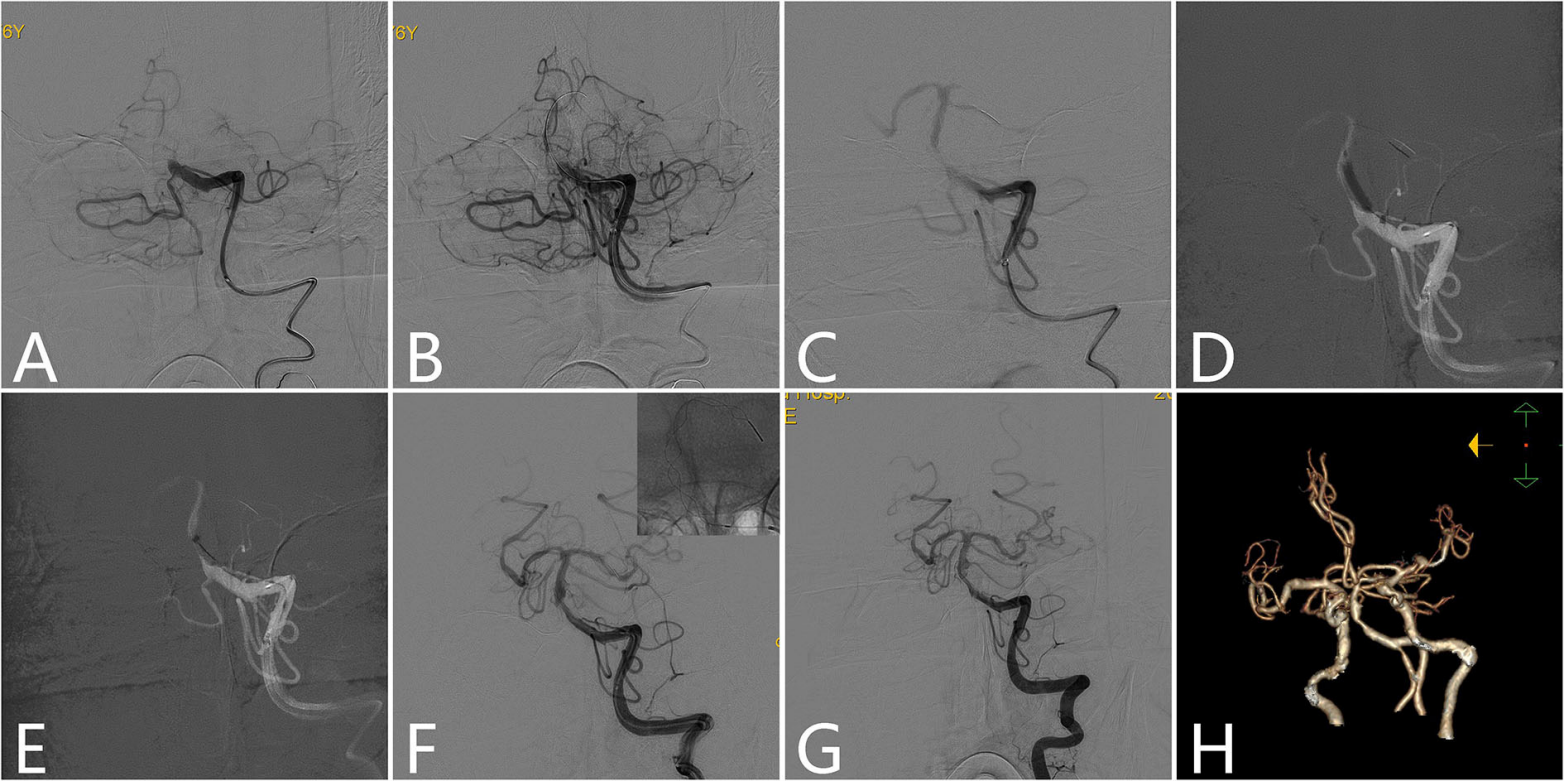
Illustrative case of BASIS technique. A 76-year-old male patient presented with dizziness for 5 h and sudden onset of unconsciousness and quadriplegia for 3 h. The admission NIHSS score was 35, and the GCS score was 5. **(A)** Left vertebral angiogram showed proximal BA occlusion. **(B)** Microcatheters and microwires completely passed through the occlusion site. Microcatheters were retrieved to the proximal segment of the occlusion site while microwires were kept in place, and angiograms showed slow blood flow through the occlusion site, that is there is a microcatheter first-pass effect phenomenon. **(C)** A Syphonet 4–30 mm stent was unsheathed at the occlusion site while the distal stent was placed at the SCA. **(D)** A Maverick 2.5/15 mm balloon was advanced over the wire of the retrieval stent into the stenotic site and partially retrieve the stent, then balloon angioplasty was performed. **(E)** The aspiration catheter was passed although the stenotic site after balloon angioplasty, and then the stent was retrieved by manual operation with a 50-ml locked syringe. **(F)** The retrieval stent was redeployed at the lesion site while the distal stent was placed at the posterior cerebarl artery (PCA). Then, the aspiration catheter was retrieved to the proximal stenotic site. An angiogram showed moderate stenosis at the proximal site of the BA, and successful reperfusion was achieved. **(G)** Sheathing and withdrawal of the retrieval stent and microwire were put through the lesion site. Twenty minutes later, a left vertebral angiogram showed that the stenosis was stable, and successful reperfusion was maintained. **(H)** Postprocedure CTA showed moderate stenosis located proximal to the BA. NIHSS, National Institutes of Health Stroke Scale; GCS, Glasgow coma scale; SCA, superior cerebellar artery; BA, basilar artery; CTA, CT angiography.

A total of seven patients (four men and three women) with acute ICAS-related LVO who received the BASIS technique (illustrated case is shown in Figure 2) were identified from our prospective acute ischemic stroke database of patients who received EVT. The patients' baseline characteristics and clinical outcomes are shown in Table 1. Six patients had hypertension, one had hyperlipidemia, one had diabetes mellitus, one had an ischemic stroke, two were smokers, two had hyperlipidemia, and none had atrial fibrillation. The median admission NIHSS score was 15 (ranging from 3 to 35), and the median baseline ASPECT score was 9 (ranging from 3 to 10). The premorbid mRS was 0 in all except 1 (mRS 2) patient. None of the patients had sICH or re-occlusion post-procedure. Two patients underwent the high-resolution MR, and irregular arteriosclerotic plaques of various thicknesses were observed on volume isotropic turbo spin echo acquisition images in these two cases. A good prognosis was observed in three patients and a poor prognosis in three patients, and one patient died because of a large infraction due to poor collateral.

**Table 1:** Summary of patient characteristics and clinical and radiological outcomes.

**Item**	
Sex ratio (male/female)	4/3
Age (year), median (range)	76 (46–90)
Admission NIHSS, median (range)	15 (3–35)
Baseline ASPECT, median (range)	9 (5–10)
Risk factors, *n* (%)	
HT	6 (85.7%)
DM	1 (14.3%)
IS	1 (14.3%)
AF	0 (0%)
Smoker	2 (28.6%)
Hyperlipidemia	2 (28.6%)
90 days mRS, *n* (%)	
0–2	4 (57.1%)
3–5	2 (28.6%)
6	1 (14.3%)
sICH, *n* (%)	0
Culprit Artery, *n* (%)	
M1-MCA	4 (57.1%)
M2-MCA	1 (14.3%)
BA	1 (14.3%)
VA intracranial	1 (14.3%)
PRT, min, median(range)	51 (35–57)
eTICI, *n* (%)	
Grade 3	7 (100%)
Device used in procedure	
Maverick Balloon	7 (100%)
Syphonet 4–30 Retrieval stent	7 (100%)
FPE, *n* (%)	7(100%)
RSR median (range)	5% (5–10%)
Procedure related complication, *n* (%)	
Distal emboli, *n* (%)	0 (0%)
Arterial dissection, *n* (%)	0 (0%)
Reocclusion, *n* (%)	0

NIHSS, National Institutes of Health Stroke Scale; ASPECT, Alberta Stroke Program Early CT Score; HT, hypertension; DM, diabetes mellitus; AF, arterial fibrillation; IS, ischemic stroke; mRS, modified Rankin Scale; sICH, symptomatic intracranial hemorrhage; MCA, middle cerebral artery; BA, basilar artery; VA, vertebral artery; eTICI, extended thrombolysis in cerebral infarction; PRT, puncture-to-recanalization time; FPE, first-pass effect; RSR, residual stenosis rate.

Radiological assessment, endovascular treatment, and outcomes are shown in Table 1. Occlusions detected were as follows: M1 segment of the middle cerebral artery (MCA) in four patients, M2-MCA in one patient, an intracranial segment of vertebral artery (VA) in one patient, and basilar artery (BA) in one patient. The median puncture-to-reperfusion time (PRT) was 51 (ranging from 35 to 57). A 0.060-inch Catalyst (Boston, USA) aspiration catheter, Maverick balloon (Boston, USA), and Syphonet (Achieva, China) retrieval stent were adopted in all patients. All patients achieved eTICI grade 3 reperfusion and FPE, none had distal emboli during the procedure, and the residual stenosis rate ranged from 5 to 10%.

## Discussion

Intracranial atherosclerosis is common in Asian countries (18), so ICAS-related acute occlusion is also high in Asia. Due to different stenosis grades, plaque characteristics, thrombus compositions, and complex angioarchitectures, the pathogenesis of stroke caused by ICAS is diverse, and EVT for ICAS is complex. Thus far, the optimal EVT strategy is uncertain. Recently, the “Stent-Pass-Aspiration-resCuE-Micowire-Angioplasty (SPACEMAN)” technique was proposed by the China Stroke Team for ICAS-related LVO (19). However, neuro-interventionists may encounter some difficulties when they adapt this technique. First, the aspiration catheter may not totally penetrate the occlusion site because of the stenotic site. Second, if the thrombus located at the distal end is not completely cleared away, the thrombus may migrate into the distal artery when the aspiration is retrieved.

The BASIS technique we propose can overcome the difficulties encountered with SPACEMAN. First, the possibility of aspiration catheters going through the occlusion site increases after balloon angioplasty at the stenotic site. Second, the retrieval stent is repositioned in the culprit artery, and the possibility of migration of the thrombus at the lesion site decreases. Third, this technique increases the safety of EVT for ICAS-related LVO. The whole EVT routine system will be more stable if the balloon advances through the wire of the retrieval stent than if the system involves the balloon advancing through a single microwire. The possibility of vessel perforation caused by microwires may increase (20).

There are some advantages of the BASIS technique in treating ICAS-related LVO. First, emergent balloon angioplasty and clot retrieval were performed simultaneously to decrease procedure-related complications (11, 12) and time-saving. Second, due to the special pathology of ICAS-related LVO, emergent angioplasty *via* balloon and/or stent was needed (21, 22). Therefore, the BASIS technique may increase the rate of successful reperfusion. Third, the possibility of vessel injury was reduced. Retrieval stent thrombectomy may cause vessel injury, especially in ICAS cases (23–25). In the BASIS technique, retrieval stent thrombectomy performed following aspiration catheter totally penetrates the stenotic lesion after balloon angioplasty. Such a procedure would minimize the touch area of the stent and vessel, especially if they did not touch the stenotic lesion, so it would minimize the possibility of vessel injury. Fourth, it is time-saving. This technique would minimize exchange passes, for example, if emergent angioplasty *via* balloon or/and the stent is needed after one pass of stent thrombectomy.

Some details should be noted in the BASIS technique. First, the most suitable case is a large clot located distal to the stenotic site, and the microcatheter first-pass effect could help neuro-interventionists recognize such lesions (14). Second, some specific characteristics of the device used in this technique are needed. The intrinsic appeal of the device is the compatibility between the stent and the balloon. The inner diameter of most balloons used in neuro-disease is 0.0165 inch or 0.017 inch (26), and the inner lumen of the balloon can allow partially retrieve of the stent. Therefore, the maximum diameter of the wire of the retrieval stent is 0.015 inch, indicating, as best we know, a retrieval stent such as Trevo XP (27) (StryKer), Syphonet (Achieva). Third, balloon angioplasty should be done with caution if the stenotic lesion covers the perforators of the penetrating artery, such as the lenticulostriate artery or pontine perforating arteries. Emergent angioplasty may cause perforating artery occlusion due to plaque or thrombus. Fourth, hyperperfusion may occur once stenosis of the culprit artery is resolved (28). Therefore, we should give attention to the phenomena that might suggest disruption of cerebral autoregulation, such as the increased diameter of arterial branches, prominent capillary blush and early draining veins of the treated territories. If such angiographic characteristics are detected, we should control blood pressure according to middle cerebral artery mean flow velocity measurements by transcranial Doppler (TCD) (29). Re-occlusion occurrence is not rare in ICAS cases that achieve successful recanalization after endovascular therapy, especially in cases with high residual stenosis (30). Therefore, it would be better to face the risk of hyperperfusion which clinicians may control by post-procedure management than the risk of re-occlusion that may need another EVT.

This study had some limitations. First, it was a single-center study with relatively few study samples. However, our study focuses on innovative endovascular strategies for treating ICAS-related LVO. Also, due to few study samples and a 100% technique success rate, we cannot find out the satiation that the BASIS technique fails which needs a large and multi-center study sample to test and modify this technique. Second, the diagnosis of ICAS was not totally confirmed because it was in an emergent setting. However, our study was performed in a high-volume stroke center and had more than 500 thrombectomy cases per year. The neuro-interventionists had enough clinical experience to distinguish ICAS from embolic cases on the basis of clinical presentation, the morphology of CTA and DSA, and the microcatheter first-pass effect, and could ensure the diagnostic accuracy of ICAS to the greatest extent. Retrieval stents and balloons are highly selected in the BASIS technique, and this may influence the generalization of this technique; however, the situation may change as the neuro-intervention device becomes highly developed, the manufacturer can produce low-profile retrieval stent which is comparable to more delicate microcatheter and which also allow balloon fully withdraw, also we can produce balloon with a large inner core which can withdraw now available retrieval stent or so on.

## Conclusion

Our case series suggests that the BASIS technique for treating acute ICAS-related LVO is technically feasible and safe and may be associated with decreasing procedure-related complications and time savings. This technique should be considered in cases of acute ICAS-related LVO, especially in cases with thrombi distal to the stenotic site. However, our findings should be confirmed by future multicenter, large-sample studies.

## Data availability statement

The raw data supporting the conclusions of this article will be made available by the authors, without undue reservation.

## Ethics statement

The studies involving human participants were reviewed and approved by Zhangzhou Municipal Hospital Ethics Committee. Written informed consent for participation was not required for this study in accordance with the national legislation and the institutional requirements.

## Author contributions

T-yY: conception and design, drafting the article, and revising it critically for important intellectual content. W-hC: conception and design, revising it critically for important intellectual content, final approval of the version to be published, and agreed to be accountable for all aspects of the work in ensuring that questions related to the accuracy or integrity of any part of the work are appropriately investigated and resolved. Y-mW: conception and design, analysis and interpretation of data, and agreed to be accountable for all aspects of the work in ensuring that questions related to the accuracy or integrity of any part of the work are appropriately investigated and resolved. D-lL, Z-nP, X-fZ, JG, and M-hW: analysis and interpretation of data, and agreed to be accountable for all aspects of the work in ensuring that questions related to the accuracy or integrity of any part of the work are appropriately investigated and resolved. X-hL, R-cC, and L-sZ: acquisition of data, analysis and interpretation of data, and agreed to be accountable for all aspects of the work in ensuring that questions related to the accuracy or integrity of any part of the work are appropriately investigated and resolved. All authors contributed to the article and approved the submitted version.

## Funding

The article is sponsored by National Health commission capacity building and continuing education center GWJJ2021100203 and Beijing Health Promotion Association: Research on Appropriate Intervention Technique for High-risk Crowds of Stroke, BHPA2021IN002.

## Conflict of interest

The authors declare that the research was conducted in the absence of any commercial or financial relationships that could be construed as a potential conflict of interest.

## Publisher's note

All claims expressed in this article are solely those of the authors and do not necessarily represent those of their affiliated organizations, or those of the publisher, the editors and the reviewers. Any product that may be evaluated in this article, or claim that may be made by its manufacturer, is not guaranteed or endorsed by the publisher.
